# Natural Language Processing for Clinical Laboratory Data Repository Systems: Implementation and Evaluation for Respiratory Viruses

**DOI:** 10.2196/44835

**Published:** 2023-06-06

**Authors:** Elham Dolatabadi, Branson Chen, Sarah A Buchan, Alex Marchand Austin, Mahmoud Azimaee, Allison McGeer, Samira Mubareka, Jeffrey C Kwong

**Affiliations:** 1 Vector Institute Toronto, ON Canada; 2 School of Health Policy and Management, Faculty of Health York University Toronto, ON Canada; 3 Institute of Health Policy, Management and Evaluation University of Toronto Toronto, ON Canada; 4 ICES Toronto, ON Canada; 5 Public Health Ontario Toronto, ON Canada; 6 Dalla Lana School of Public Health University of Toronto Toronto, ON Canada; 7 Sinai Health System Toronto, ON Canada; 8 Department of Laboratory Medicine and Pathobiology University of Toronto Toronto, ON Canada; 9 Sunnybrook Research Institute Toronto, ON Canada; 10 University Health Network Toronto, ON Canada; 11 Department of Family and Community Medicine University of Toronto Toronto, ON Canada

**Keywords:** health, informatics, natural language processing, knowledge extraction, electronic health record, EHR

## Abstract

**Background:**

With the growing volume and complexity of laboratory repositories, it has become tedious to parse unstructured data into structured and tabulated formats for secondary uses such as decision support, quality assurance, and outcome analysis. However, advances in natural language processing (NLP) approaches have enabled efficient and automated extraction of clinically meaningful medical concepts from unstructured reports.

**Objective:**

In this study, we aimed to determine the feasibility of using the NLP model for information extraction as an alternative approach to a time-consuming and operationally resource-intensive handcrafted rule-based tool. Therefore, we sought to develop and evaluate a deep learning–based NLP model to derive knowledge and extract information from text-based laboratory reports sourced from a provincial laboratory repository system.

**Methods:**

The NLP model, a hierarchical multilabel classifier, was trained on a corpus of laboratory reports covering testing for 14 different respiratory viruses and viral subtypes. The corpus includes 87,500 unique laboratory reports annotated by 8 subject matter experts (SMEs). The classification task involved assigning the laboratory reports to labels at 2 levels: 24 fine-grained labels in level 1 and 6 coarse-grained labels in level 2. A “label” also refers to the status of a specific virus or strain being tested or detected (eg, influenza A is detected). The model’s performance stability and variation were analyzed across all labels in the classification task. Additionally, the model's generalizability was evaluated internally and externally on various test sets.

**Results:**

Overall, the NLP model performed well on internal, out-of-time (pre–COVID-19), and external (different laboratories) test sets with microaveraged *F*_1_-scores >94% across all classes. Higher precision and recall scores with less variability were observed for the internal and pre–COVID-19 test sets. As expected, the model’s performance varied across categories and virus types due to the imbalanced nature of the corpus and sample sizes per class. There were intrinsically fewer classes of viruses being detected than those tested; therefore, the model's performance (lowest F_1_-score of 57%) was noticeably lower in the detected cases.

**Conclusions:**

We demonstrated that deep learning–based NLP models are promising solutions for information extraction from text-based laboratory reports. These approaches enable scalable, timely, and practical access to high-quality and encoded laboratory data if integrated into laboratory information system repositories.

## Introduction

Clinical laboratory data account for a large proportion of data stored in electronic health record systems worldwide and present a wealth of information vital for evidence-based decision-making and public health improvement [1,2]. Laboratory information systems record, manage, and store laboratory test data to facilitate reporting to clinicians and jurisdictional laboratory information repositories [3]. These repositories often include test orders and results from various laboratory service providers, such as hospitals, public health agencies, and private companies, and are populated as part of clinical care.

Several factors limit the secondary use of laboratory data for other purposes. The most important are concerns about the quality of the data, lack of standardization, and difficulty extracting the needed information [4,5]. Laboratory data vary over time due to evolving standards of care and changing population demographics. Furthermore, specific categories of laboratory data are reported as free text in an unstructured format with no standard vocabulary in the actual contents, which adds more complexity for their secondary uses [1]. Therefore, efforts are needed to eliminate redundancies, extract the necessary information, and derive accurate interpretations from laboratory data.

**Figure 1:**
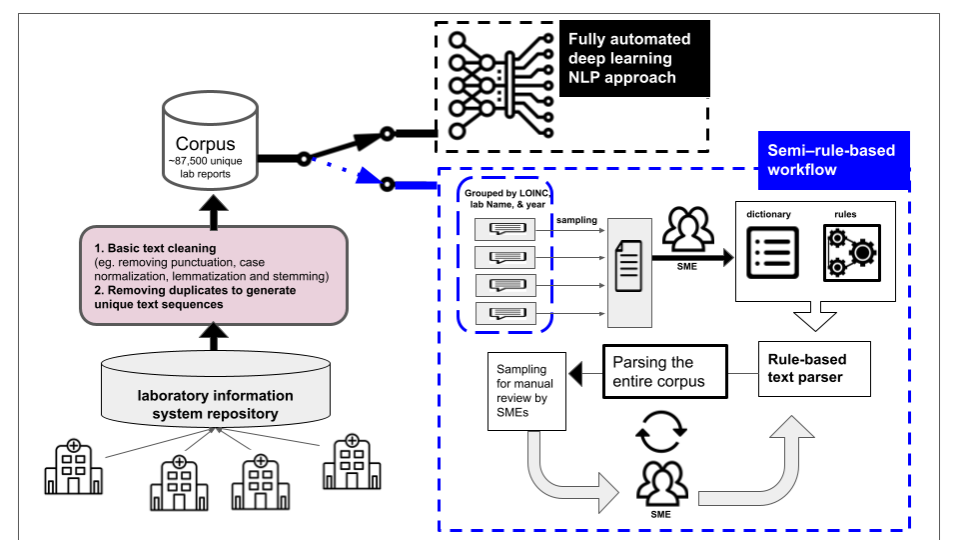
Semi–rule-based workflow versus fully automated deep learning natural language processing (NLP) approach. Semi–rule-based relies on time-consuming and operationally resource-intensive approaches for the information extraction task. The corpus was derived from the Ontario Laboratories Information System (OLIS). Following basic text-cleaning steps, around 87,500 unique laboratory reports were collected and included in our corpus to be used in parallel by both semi–rule-based and deep learning NLP approaches. Semi–rule-based workflow is a multistep procedure where all the unique reports were grouped by Logical Observation Identifiers Names and Codes (LOINC), year, and location in the first step. In the second step, subject matter experts (SMEs) created a list of dictionaries for terms related to the different viruses and strains and a set of if-then-else rules to generate interpretations and extract information from each laboratory report. The dictionaries and if-then-else rules were packaged as a python library called the rule-based text parser. Finally, the parser was improved based on inputs from 3 SMEs in an iterative manner.

Our institute, ICES, has developed a specific information extraction workflow to manage the interpretation of a large volume of provincial clinical laboratory results, as shown in Figure 1. The workflow, called a semi–rule-based workflow, relies on time-consuming and operationally resource-intensive approaches, including a library of rule-based and handcrafted tools. These tools are explicitly programmed for various laboratory result categories and must be refined continually. To address challenges with our existing semi–rule-based workflow and automate the exhaustive information retrieval task, we built a deep learning–based natural language processing (NLP) tool. The objective of this study was to assess the feasibility of our deep learning–based NLP model and evaluate its performance relative to the semi–rule-based workflow.

The development of NLP methods is essential to automatically transform laboratory reports into a structured representation that scales data usability for research, quality improvement, and clinical purposes [6-12]. NLP enables automated extraction of information, and its use in the clinical domain is growing, with increasing uptake in various applications such as biomedical named entity recognition [11,12], summarization [10], and clinical prediction tasks [9]. More recently, deep learning approaches such as convolutional neural networks, recurrent neural networks (RNNs), and RNN variants such as bidirectional long short-term memory (Bi-LSTM) have been successfully applied to clinical NLP tasks [10,13-16]. They are now considered the baseline techniques for various information extraction tasks [11,12,17-20].

In this study, we focused on automating the retrieval of information related to respiratory viruses from the laboratory repository of Ontario, Canada’s most populous province. Respiratory viruses account for a substantial burden of disease globally [21,22], causing both respiratory and nonrespiratory illnesses [23]. It is impossible to distinguish which respiratory virus is causing infection based on clinical examination alone, necessitating laboratory testing for confirmation. We sought to (1) implement a deep learning–based NLP predictive model to extract respiratory virus information from the laboratory repository and (2) evaluate the generalizability and robustness of predictions (extracted information) across different categories of respiratory viruses and test sets. Our study findings can inform public health practitioners and researchers about using NLP approaches to empower and facilitate access and retrieval of information from a collection of text-based laboratory reports without any time-consuming handcrafted rule-based approaches. This can facilitate the development of a scalable and easily deployable automated information extraction tool.

## Methods

### Study Design

The data set used in this study was a collection of laboratory reports that covered testing for 14 different respiratory viruses and viral subtypes (Table 1), most of which were in the form of texts. The reports were text-based and required cleaning, parsing, and encoding.

The data set was derived from the Ontario Laboratories Information System (OLIS). OLIS has over 100 contributors, which comprise hospital, commercial, and public health laboratories, adding to the complexity and variability of the clinical data. These data were analyzed at ICES.

**Figure 2:**
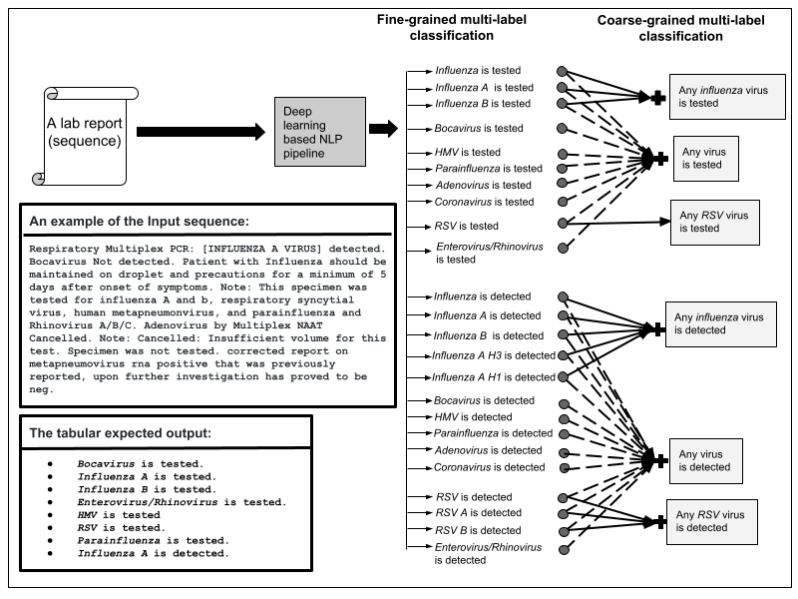
The fully automated deep learning–based natural language processing (NLP) approach is a hierarchical-based multilabel classification task that retrieves virus (or strain) types and identifies their status as being tested and/or detected. Note that a sequence refers to the input laboratory reports to the NLP approach, which may be a single or several sentences. A label also refers to the status of a specific virus or strain (tested or detected). “influenza is tested” implies it was tested for any influenza type; however, the total number of “influenza is tested” is greater than the total number of “influenza A tested + influenza B tested” since not all influenza types are mentioned. The same applies to “influenza is detected” and “RSV is tested.” HMV: human metapneumovirus; NAAT: nucleic acid amplification test; PCR: polymerase chain reaction; RSV: respiratory syncytial virus.

The automated encoding of laboratory testing reports into respiratory viruses is framed as a multilabel hierarchical classification task to address the needs of knowledge users in our institute in distinguishing respiratory viruses. According to our users, information at 2 resolution levels is needed: high and low. Therefore, we defined 2 levels of a classification hierarchy, and at each level, the classification was multilabel. Each input text sequence was assigned to a nonempty subset of various labels, as shown in Figure 2. In the first level of the hierarchy, the classifier assigned outputs to 24 mutually nonexclusive fine-grained labels. The fine-grained labels were reassigned to 6 coarse-grained sets of labels in the second level of the classification hierarchy. In this work, “sequence” refers to the input laboratory reports to the NLP model, which may be single or several sentences. A “label” also refers to a status of a specific virus or strain being tested or detected.

To summarize, the information extraction for an input text sequence involved retrieving virus types and identifying their status as being tested and/or detected. Figure 2 illustrates a running example of the input and output of the deep learning–based NLP model.

**Table 1:** Details of the respiratory viruses embedded in text-based laboratory reports derived from the Ontario Laboratories Information System (OLIS). Specimens may be tested for 1 or more of the following viruses: influenza, RSV^a^, adenoviruses, seasonal coronaviruses, enterovirus/rhinoviruses, parainfluenza viruses, HMV^b^, and bocavirus^c^.

Viruses	Mention counts^d^, n (%)	Tested^e^, n (%)	Detected^f^, n (%)
Adenovirus	21,614 (7)	45 (6)	2 (1)
Bocavirus	5112 (2)	96 (13)	5 (3)
Coronavirus (seasonal)	9128 (3)	95 (13)	9 (5)
Any influenza	49,282 (16)	78 (11)	35 (20)
Influenza A	44,753 (15)	80 (11)	30 (18)
Influenza A H1	6797 (2)	N/A^g^	17 (10)
Influenza A H3	9929 (3)	N/A	18 (10)
Influenza B	40,840 (13)	78 (11)	12 (7)
Enterovirus/rhinovirus	13,262 (4)	92 (13)	19 (11)
HMV	21,194 (7)	46 (6)	3 (2)
Parainfluenza	21,584 (7)	46 (6)	4 (2)
Any RSV	38,080 (12)	68 (9)	11 (6)
RSV A	11,227 (4)	N/A	2 (1)
RSV B	11,094 (4)	N/A	3 (2)
Total	303,896 (100)	724 (100)	170 (100)

^a^RSV: respiratory syncytial virus.

^b^HMV: human metapneumovirus.

^c^The testing modalities employed include single and multiplex polymerase chain reaction (PCR), direct fluorescent antibody, viral culture, and enzyme immunoassay rapid antigen tests. Repeated testing may involve multiple laboratories and testing modalities.

^d^Represents the counts of specific virus terms from all the distinct laboratory reports (unique sequences). It does not provide any clinical information regarding the prevalence of the aforementioned viruses in Ontario.

^e^Represents the proportion of mentions flagged as tested by the parser.

^f^Represents the proportion of mentions flagged as positively detected by the parser. Note that tested and detected are not mutually exclusive; we first determined whether it was tested for (ie, has e a result) and then flagged it as detected if the result is positive. Detected is a subset of the tested.

^g^N/A: not applicable. Note that the subtypes of influenza A and RSV were only analyzed for detection but not testing, as the scope of the planned analyses for using the respiratory virus data was primarily focused on the larger virus categories.

### Corpus Development Description

#### About OLIS

To create the corpus for this study, over a million observations corresponding to 99 unique Logical Observation Identifiers Names and Codes (LOINC) were pulled from OLIS, and the text-based laboratory results were extracted from the observations. OLIS was created and is managed by Ontario Health, from whom ICES receives an ongoing data feed. At the time of writing this paper, the OLIS data held at ICES consists of >9000 unique LOINC and >5 billion laboratory observations across 150 laboratory test centers in Ontario. As such, the clinical laboratory data have considerable complexity and variability.

#### Development of the Ground Truth

In this study, we leveraged the semi–rule-based workflow, an information extraction workflow relying on a rule-based and handcrafted tools library, to create ground truth for the deep learning model. A group of 8 SMEs was engaged in performing the required tasks in the workflow; they comprised 2 infectious disease epidemiologists (authors JCK and SAB), 2 infectious disease microbiologists (AM and SM), a genomic specialist (AMA), a research methodologist (MA), a data analyst (BC), and a machine learning scientist (ED). These tasks included basic text cleaning, quality checking, and rule-based algorithm development for interpreting reports, as shown in Figure 1. In our institute, LOINC are mainly used to filter OLIS observations into relevant groupings (eg, respiratory viruses) and not for encoding and interpretation since they are not always used appropriately by those entering the data into OLIS. Consequently, the SMEs identified a list of 99 LOINC related to respiratory viruses, and all the laboratory reports in OLIS corresponding to these LOINC were retrieved. The workflow consists of 3 tasks, which are detailed in the subsequent paragraphs.

First, the data analyst and data scientist (authors BC and ED) scanned the text strings. After performing basic text cleaning (eg, removing punctuations, stop words, case normalization, lemmatization, and stemming) and removing duplicates, they created a meaningful list of 87,500 unique laboratory reports.

Next, the unique reports were grouped by laboratory and facility names, LOINC, and year. Then, 3 SMEs, including 2 analysts and an infectious disease specialist, manually reviewed multiple samples per group and created a knowledge base and sets of if-then-else rules to generate interpretations for each laboratory report. Specifically, the knowledge base consisted of dictionaries for terms related to the different viruses and strains. The if-then-else rules provided instructions for grouping virus terms with respective results packaged as a Python library, which we refer to in this study as the rule-based text parser.

Following the initial development of the rule-based text parser, it was improved based on inputs from 3 other SMEs in an iterative manner. The text parser was applied to the entire corpus to generate annotations at each iteration. Next, the data analyst manually reviewed the interpretations and flagged unclear results to be reviewed by SMEs at another iteration. In addition, a small random sample of unflagged test results was provided to SMEs to be reviewed at this iteration. The SMEs subsequently reviewed the list and provided new rules to be added to the text parser. This procedure was repeated until there were no more flagged test results.

### Model Development and Evaluation

#### NLP Model Description

The deep learning–based NLP model consisted of 3 components that were trained jointly: the word embedding layer, the Bi-LSTM layer, and the output layer. The word embedding layer computed a vector representation of each word in the text as a combination of a character-based representation learning model [24,25] and word vectors initialized with pretrained global vectors (GloVe) embeddings [26]. The embedding layer was coupled with a Bi-LSTM on top of it to generate conceptually and contextually meaningful representations of words. An output layer of a size equal to the number of distinct labels was placed on top of Bi-LSTM, and the last hidden state of the Bi-LSTM was projected into the output layer.

#### Model Evaluation

The model’s robustness and generalizability were evaluated internally and externally on various test sets, as shown in Table 2. The internal test set used for model training was a randomly sampled subset representing 10% (n=6719) of the laboratory reports from OLIS from 2007 to 2018. The performance of the model was also evaluated on 2 out-of-time test sets, including samples from an entirely different time period: (1) a large pre–COVID-19 (2019) sample and (2) a small post–COVID-19 (2020) sample. A separate test set, denoted as the external test set, included samples up to 2019 from 2 separate laboratories (testing sites not included in the development of the model) and was used to assess the external generalizability of the model. *F*_1_-scores, along with precision and recall scores, were calculated for the model’s predictions. A 2-tailed paired *t* test was used to determine whether there was a statistically significant difference in the *F*_1_-scores between classes and test sets. In addition, 95% CIs were calculated for the precision and recall scores to quantify the uncertainty of the model's estimates.

**Table 2:** Data set statistics for laboratory descriptions of the development and test sets.

Cohorts		Sequences^a^, n (%)	Any influenza virus^b^		Any RSV^c^ virus		Any virus	
Total		87411 (100%)	Detected, n (%)	Tested, n (%)	Detected, n (%)	Tested, n (%)	Detected, n (%)	Tested, n (%)
**Development set (2009-2018)**								
	Training set	60,471 (69)	13,792 (16)	35,292 (40)	3959 (4)	27,196 (31)	22,284 (25)	40,652 (46)
	Internal test set	6719 (8)	1604 (2)	3941 (4)	428 (0.5)	3009 (3)	2541 (3)	4534 (5)
**Out-of-time test sets**								
	Pre–COVID-19 (2019)	15,908 (18)	3019 (3)	6903 (8)	706 (0.8)	5957 (7)	4745 (5)	8643 (10)
	Post–COVID-19 (2020)	100 (0.01)	N/A^d^	11 (0.01)	<6 (0.006)	11 (0.01)	<6 (0.006)	27 (0.03)
External test set (2009-2018)		4213 (5)	864 (1)	3020 (34)	261 (0.2)	2546 (3)	1431 (2)	3237 (4)

^a^Represents the counts of unique sequences; a sequence refers to the input laboratory reports to the NLP model, which may be a single sentence or several sentences.

^b^Detected and tested represent the aggregation of the proportion of any mentions of the virus terms from the total unique sequences in the data set.

^c^RSV: respiratory syncytial virus.

^d^N/A: not applicable.

### Ethical Considerations

The use of the data in this study was approved by the ICES Privacy and Legal Office. Projects that solely use data collected by ICES under section 45 of Ontario’s Personal Health Information Protection Act (PHIPA) are exempt from research ethics board review. Section 45 of the PHIPA authorizes ICES to collect personal health information, without consent for the purpose of analyzing or compiling statistical information concerning the management, evaluation, monitoring, and allocation of resources to or planning for the health system.

## Results

**Figure 3:**
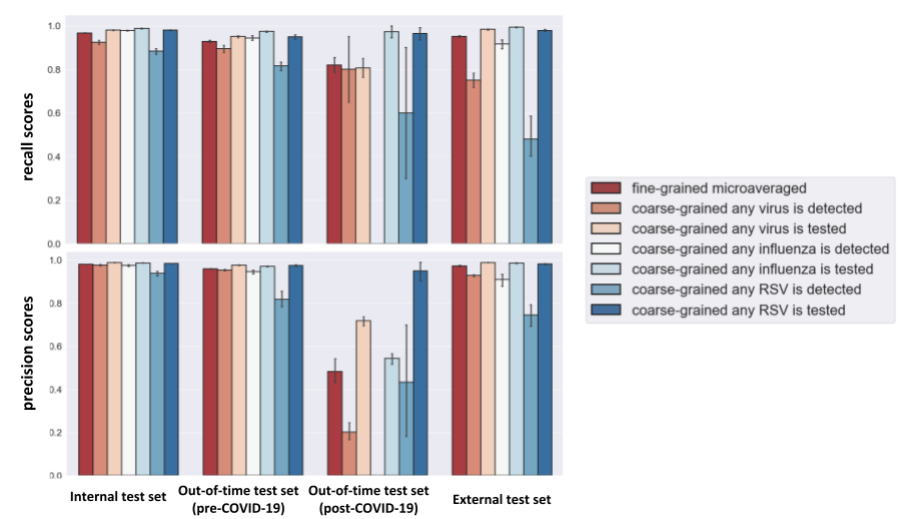
The precision and recall scores of the predictions of the top 10 best-performing models with 95% CIs. The fine-grained results are aggregated by microaveraging across 24 fine-grained labels. RSV: respiratory syncytial virus.

The development corpus, including training and test sets, included 87,500 sequences involving ~5 million tokens. The summary statistics for the data sets are shown in Table 2. The NLP model was implemented in TensorFlow on an NVidia Tesla (Nvidia) graphics processing unit, and Adam optimization was used as the optimization algorithm (more details in Multimedia Appendix 1). The maximum sequence length was fixed to 400 words. The model was trained several times with random initialization on the development corpus, and the results of the top 10 best-performing models on the test sets are presented in this paper. The results for the fine-grained classification in the first level of the hierarchy are presented in Table 3 and aggregated by microaveraging across the 24 fine-grained labels. Detailed performance for each label is also shown in Multimedia Appendix 2. The *F*_1_-score performance of the model in the second level of the hierarchy, coarse-grained multilabel classification, for “any influenza,” “any RSV” (respiratory syncytial virus), and “any virus” are shown in Table 3. In addition, the variation of the model’s precision and recall scores using bar plots and 95% CIs are shown in Figure 3.

As expected, the performance on the internal test set was better than the out-of-time (pre–COVID-19) and external test sets. In this regard, the *F*_1_-score results of the test sets were compared, and noticeable differences were observed between the pairs of internal and out-of-time (pre–COVID-19) test sets, internal and out-of-time (post–COVID-19) test sets, and internal and external test sets. The out-of-time (post–COVID-19) test set was a small and imbalanced sample, including 100 sequences with <6 mentions of any virus as being detected. The sample included 12 sequences labeled as being tested for coronavirus, and our model correctly classified them with an *F*_1_-score of 0.67. Regarding the degree of uncertainty in the estimates, fewer variations in precision and recall scores are observed for the internal and out-of-time test sets (pre–COVID-19). On the contrary, the estimates on the out-of-time (post–COVID-19) and external test sets have larger CIs.

In general, the models’ estimates on any test sets were variable across classes with varying degrees of uncertainty. The averaged *F*_1_-scores of the estimates for both fine-grained (microaveraged) and “coarse-grained any virus” classes were above 90% on the internal test set. The *F*_1_-score for the “coarse-grained any influenza detected” on all test sets was above 91%. Overall, the performance for coarse-grained detected classes was lower than for coarse-grained tested classes. Among the detected classes, the performance for “any influenza virus” was evidently higher than “any RSV virus.” The same result was observed between “any influenza virus” and “any RSV virus.” Comparably, larger CIs are evidenced for the “coarse-grained any RSV detected” estimates.

**Table 3:** The prediction results (*F*_1_-score) of the top 10 best-performing models on the in-time, out-of-time, and external test sets. The fine-grained results are aggregated by microaveraging across 24 fine-grained labels.

Variables		Internal test set	Out-of-time test set^a^		External test set
			(Pre–COVID-19)	(Post–COVID-19)	
Fine-grained microaveraged, mean (SD)		97.3 (0.25)	94.31 (0.59)	60.45 (7.99)	96.23 (0.38)
**Coarse-grained any influenza virus, mean (SD)**					
	Detected^b^	97.64 (0.28)	94.47 (1.04)	N/A^c^	91.11 (2.14)
	Tested^b^	98.71 (0.15)	97.26 (0.45)	69.8 (4.43)	98.94 (0.1)
**Coarse-grained any RSV^d^, mean (SD)**					
	Detected	90.94 (1.7)	81.56 (3.63)	48.33 (44.76)	57.68 (12.53)
	Tested	98.16 (0.34	96.18 (0.95	95.6 (5.69)	98.02 (0.47)
**Coarse-grained any virus, mean (SD)**					
	Detected	95.01 (1)	92.31 (1.59)	31.71 (9.44)	82.83 (3.27)
	Tested	98.4 (0.17	96.3 (0.35)	75.87 (4.82)	98.59 (0.2)

^a^The out-of-time test set (post–COVID-19) is a very small and imbalanced sample, including only 100 sequences with no mentions of any virus detected.

^b^Detected and tested represent the aggregation of the proportion of any mentions of the virus terms from the total unique sequences in the data set.

^c^N/A: not applicable.

^d^RSV: respiratory syncytial virus.

## Discussion

### Principal Findings

In this study, we demonstrated an implementation and evaluation of an NLP model for an automated and reductive information extraction task in a province-wide laboratory data repository. Our results suggest that the NLP model is a promising approach for information extraction from text-based laboratory reports as an alternative method to address the time-consuming and operationally resource-intensive nature of handcrafted rule-based models.

### Overview of Model Findings

#### Generalization Across Various Test Sets

Overall, the NLP solution, which was a hierarchical multilabel classifier, performed well on the internal, out-of-time (pre–COVID-19), and external (different laboratories) test sets. Except for the internal test sets, the other test sets were sourced from either a more recent time period or other laboratory sites, but the model was able to generalize well with microaveraged *F*_1_-score >94% across all classes. The performance of the model on the other out-of-time (post–COVID-19) test set was satisfactory; however, due to its small sample size with many underrepresented classes, it was not possible to draw any conclusion. The out-of-time (post–COVID-19) test set was pulled from the 2020 cohort to simulate a nonstationary production environment for observation.

#### Stability and Performance Variation Between Classes

In general, the model’s performance on any test sets was variable across classes and virus types due to the imbalanced nature of the corpus and sample sizes per class. There were intrinsically fewer classes of viruses detected compared with those tested. Therefore, the model’s performance was noticeably lower in the “detected” cases. Among the detected cases, the lowest performance was observed for RSV, and the highest performance among the tested cases was observed for influenza. Moreover, more considerable variations were observed for the positive predictive and sensitivity values of the detected classes, particularly for the “any RSV virus detected” class.

### Comparison With Prior Work

Deep learning–based NLP approaches have demonstrated efficacy in many clinical NLP tasks and have thoroughly permeated the informatics community. The existing body of literature has mainly focused on using deep learning models to extract and interpret cancer-related clinical concepts [17,27,28] from free text or other clinically meaningful entities from radiology reports or hospital notes [10,15]. At the time of writing this paper, only 1 study has explored the use of an NLP system, Topaz, for the automated extraction and classification of influenza-related terms from text emergency reports [29-31]. To our knowledge, our study is the first to explore using deep learning models for efficient processing and extraction of clinically meaningful knowledge pertaining to respiratory viruses from a laboratory repository.

One strength of the NLP approach used in this study is its scalability for various text-based laboratory scenarios. As the size and complexity of laboratory data grow, so does the need for scalable and reusable tools for automated extraction of knowledge from vast amounts of clinical notes and quick generalization from 1 task to another. Manual processing of laboratory reports severely limits the utilization of rich information embedded in the data repositories and makes the process of data cleaning and quality improvement prohibitively expensive. Usually, the rules learned from cleaning a single collection of laboratory reports show little generalizability toward other collections. On the other hand, deep learning–based NLP algorithms are well poised to scale the information extraction process. Although building deep learning–based NLP models is computationally intensive and memory demanding, the benefit-to-cost ratio of these models in clinical settings will continue to increase.

### Limitations

Although this deep learning model promises great potential for digitized health data, putting the model into production and prospectively validating operational data is as crucial as model building and a critical step in assessing and ensuring its operational effectiveness. However, we expect the model’s performance to deteriorate as it goes into production, potentially impacting data quality. Moving forward, we plan to run a silent-period production validation to further prospectively explore the model’s performance. During the silent period, our model will be integrated into the data quality and management workflow for the laboratory data repository, and the outputs will be internally validated in a fashion that would avoid exposure to data users. We also plan to run rigorous evaluation and continuous refinement of the model in the silent period to assess its performance better before it enters production. Transformers heralded a new era in the NLP field and have shown to be very successful in many tasks. Our future direction includes improving the performance of our NLP pipeline by adding transformer models.

Another significant limitation of this study is that the model was only trained on respiratory virus laboratory reports. Even within that collection, some categories were naturally underrepresented, which impacted the model's generalizability. Therefore, during the silent period, more records from a diverse set of laboratory reports from various categories will be annotated and made available to the model, and the model will be updated accordingly. Finally, this study lacks explainability, which could limit the adoption of our deep learning–based models in future applications. Therefore, we plan to develop parallel pipelines that help explain the representations of the laboratory reports and the classifier’s decision boundary. 

### Conclusion

The health industry is rapidly becoming digitized, and information extraction is a promising method for researchers and clinicians seeking quick retrieval of information embedded in texts. This study described developing and validating a deep learning–based NLP approach to extract respiratory virus testing information from laboratory reports. We demonstrated that our system could classify and encode large volumes of text-based laboratory reports with high performance without any of the previous time-consuming handcrafted feature engineering approaches. Taken together, the findings of this study provide encouraging support that NLP-based information extraction could become an important component of laboratory information repositories to assist researchers, clinicians, and health care providers with their information and knowledge management tasks.

## Appendix

Multimedia Appendix 1 Details of hyperparameter tuning.

Multimedia Appendix 2 Fine-grained classification results (F1-scores from the best performing model).

## Abbreviations

Bi-LSTM: bidirectional long short-term memory
GloVe: global vectors
LOINC: Logical Observation Identifiers Names and Codes
NLP: natural language processing
OLIS: Ontario Laboratories Information System
PHIPA: Personal Health Information Protection Act
RNN: recurrent neural network
RSV: respiratory syncytial virus
SME: subject matter expert

## Data Availability

The data underlying this work are held securely in coded form at ICES and therefore cannot be shared publicly due to data privacy concerns and legal data sharing agreements between ICES and data providers (eg, health care organizations and the government). However, data access might be granted to those who meet prespecified criteria for confidential access (email: das@ices.on.ca).
