# Supplementary material for: Natural Language Processing for Clinical Laboratory Data Repository Systems: Implementation and Evaluation for Respiratory Viruses
Source: JMIR AI. 2023 Jun 6;2:e44835. doi: 10.2196/44835 (PMC11057455; doi:10.2196/44835)
Supplement: Multimedia Appendix 1 [file ai_v2i1e44835_app1.pdf]

Multimedia Appendix 1

Details of Hyperparameter Tuning

The input embedding layer included character representation with 30 dimensions and pre-trained word embeddings with 300 dimensions together with a dimensionality of 330. The Bi-LSTM neural network NLP model had one hidden layer with spatial dropout and batch normalization. The NLP model was implemented in TensorFlow on a NVidia Tesla GPU, and Adam was used as the optimization algorithm. The maximum sequence length was fixed to 400 words, and took almost 10 hours and 200 epochs for the model to reach its highest performance on the validation set. All experiments were run several times to mitigate the effects of a random seed.
