# Supplementary material for: Natural Language Processing for Clinical Laboratory Data Repository Systems: Implementation and Evaluation for Respiratory Viruses
Source: JMIR AI. 2023 Jun 6;2:e44835. doi: 10.2196/44835 (PMC11057455; doi:10.2196/44835)
Supplement: Multimedia Appendix 2 [file ai_v2i1e44835_app2.pdf]

Multimedia Appendix 2

S Table 1 Fine-grained classification results (F1-Scores (%) from the best performing model).

Labels	In-time test set	Out-of-time test set (Pre-COVID19)	Out-of-time test set (Post-COVID19)	External test set
Adenovirus is detected	65.65	80.76	0.00	47.89
Adenovirus is tested	97.49	96.78	88.89	98.11
Bocavirus is detected	62.07	14.29	0.00	0.00
Bocavirus is tested	97.95	71.97	0.00	97.75
Coronavirus (seasonal) is detected	72.57	74.55	0.00	27.27
Coronavirus is tested	97.99	92.73	66.67	96.75
Any Influenza is detected	97.78	95.38	0.00	94.45
Any Influenza is tested	98.73	97.64	64.52	98.92
Influenza A is detected	97.50	95.68	0.00	89.47
Influenza A is tested	98.33	97.23	50.00	98.91
Influenza B is detected	94.64	68.91	0.00	53.91
Influenza B is tested	98.54	97.19	45.45	99.02
Influenza A H1 is detected	85.53	74.82	0.00	75.76
Influenza A H3 is detected	82.19	64.22	0.00	44.04
Enterovirus/Rhinovirus is detected	89.45	86.96	100.00	45.67
Enterovirus/Rhinovirus is tested	97.70	93.37	72.73	96.10
Human Metapneumovirus (HMV) is detected	85.44	78.49	0.00	60.47
Human Metapneumovirus (HMV) is tested	98.60	98.14	88.89	99.23
Parainfluenza is detected	90.08	83.20	0.00	84.30
Parainfluenza is tested	98.15	96.82	88.89	98.94
Any Respiratory Syncytial Virus (RSV) is detected	92.25	83.08	66.67	55.98
Any Respiratory Syncytial Virus (RSV) is tested	98.13	96.18	90.91	97.52
RSV A is detected	22.22	2.44	0.00	0.00
RSV B is detected	62.86	17.39	0.00	0.00
